# Relationship Between Mean Vancomycin Trough Concentration and Mortality in Critically Ill Patients: A Multicenter Retrospective Study

**DOI:** 10.3389/fphar.2021.690157

**Published:** 2021-07-19

**Authors:** Yanli Hou, Jiajia Ren, Jiamei Li, Xuting Jin, Ya Gao, Ruohan Li, Jingjing Zhang, Xiaochuang Wang, Xinyu Li, Gang Wang

**Affiliations:** ^1^ Department of Critical Care Medicine, the Second Affiliated Hospital of Xi’an Jiaotong University, Xi’an, China; ^2^ Department of Critical Care Medicine, the Second Affiliated Hospital of Xi’an Medical University, Xi’an, China

**Keywords:** vancomycin trough concentration, intensive care unit, mortality, eICU collaborative research database, observational study

## Abstract

**Background:** It remains unclear whether the mean vancomycin trough concentration (VTC) derived from the entire course of therapy is of potential benefit for critically ill patients. This study was conducted to explore the association between mean serum VTC and mortality in intensive care units (ICUs).

**Methods:** 3,603 adult patients with two or more VTC records after receiving vancomycin treatment in the eICU Collaborative Research Database were included in this multicenter retrospective cohort study. Mean VTC was estimated using all measured VTCs and investigated as a continuous and categorical variable. Patients were categorised into four groups according to mean VTC: <10, 10–15, 15–20, and >20 mg/L. Multivariable logistic regression and subgroup analyses were performed to investigate the relationship of mean VTC with mortality.

**Results:** After adjusting for a series of covariates, logistic regression analyses indicated that mean VTC, as a continuous variable, was positively correlated with ICU (odds ratio, 1.038, 95% confidence interval, [1.014–1.063]) and hospital (1.025 [1.005–1.046]) mortalities. As a categorical variable, mean VTC of 10–15 mg/L was not associated with reduced ICU (1.705 [0.975–2.981]) and hospital (1.235 [0.829–1.841]) mortalities. Mean VTC of 15–20 mg/L was not correlated with a lower risk of hospital mortality (1.370 [0.924–2.029]). Moreover, mean VTCs of 15–20 and >20 mg/L were significantly associated with higher ICU mortality (1.924 [1.111–3.332]; 2.428 [1.385–4.258]), and mean VTC of >20 mg/L with higher hospital mortality (1.585 [1.053–2.387]) than mean VTC of <10 mg/L. Similar results were observed in patients with different Acute Physiology and Chronic Health Evaluation IV score, creatinine clearance, age, and body mass index subgroups.

**Conclusion:** Mean VTC was not associated with reduced ICU/hospital related mortality. Our results suggested that VTC monitoring might not guarantee vancomycin efficacy for ICU patients.

## Introduction

Vancomycin, a glycolpeptide bactericide that acts by obstructing the synthesis of the bacterial cell wall, was isolated from streptomycin approximately half a century ago (Moellering, 2006). It is widely used to treat Gram-positive bacterial infections, including methicillin-resistant *Staphylococcus aureus* (MRSA) and exhibits time-dependent bactericidal activity with a long post-antibiotic effect (Álvarez et al., 2016; Liu et al., 2011; Rybak, 2006). Therapeutic drug monitoring (TDM) is an adjuvant and practical method used for vancomycin dosing adjustment in intensive care units (ICUs) because of its narrow therapeutic window (Tabah et al., 2015). Based on infection models and clinical pharmacokinetic/pharmacodynamic (PK/PD) studies, the area under the concentration-time curve over 24 h/minimum inhibitory concentration (AUC_0–24 h_/MIC) has been established as the most predictive index to reflect the clinical and microbiological efficacies of vancomycin (Jung et al., 2014; Moise-Broder et al., 2004). Bacterial clearance, along with improvements in clinical signs and symptoms, has been suggested to be associated with AUC_0–24h_/MIC ≥ 400 (Makmor-Bakry et al., 2019; Rybak et al., 2020). However, it is difficult to precisely determine multiple serum/tissue vancomycin concentrations during the same dosing interval to calculate the AUC in clinical practice (Rybak et al., 2009).

Serum vancomycin trough concentration (VTC) monitoring before the fourth dose has been suggested as the most practical method for TDM of vancomycin, as a VTC 15–20 mg/L may achieve an AUC_0–24h_/MIC ≥400 if the MIC is ≤ 1 mg/L (Rybak et al., 2009). The Infectious Diseases Society of America has recommended that the VTC should be maintained above 10 mg/L to avoid the development of resistance, and 15–20 mg/L to improve clinical outcomes (Rybak et al., 2009). The Chinese Pharmacological Society recommended 10–15 mg/L as the target VTC for adult patients, and 10–20 mg/L for adult patients with severe MRSA infections (Ye et al., 2016). However, plenty of studies have demonstrated that VTC did not have a considerable effect on treatment outcomes (Albur et al., 2012; Jung et al., 2014; Liang et al., 2018; Makmor-Bakry et al., 2019) and highlighted a high incidence of inappropriate VTC leading to increased healthcare costs (Seng et al., 2018). As a result, the latest consensus in 2020 advocated vancomycin area under the concentration-time curve (AUC) values obtained by Bayesian modelling as the most accurate approach for managing vancomycin dosing (Rybak et al., 2020). Nevertheless, to our knowledge, those most retrospective and prospective studies only focused on the initial VTC after vancomycin therapy. It remains unclear whether the mean VTC, estimated using all measured VTCs during the entire course of treatment, is beneficial for critically ill patients. Therefore, this multicenter observational study with a large sample was further performed to investigate the association of mean VTC with mortality in critically ill patients.

## Materials and Methods

### Data Source and Study Design

This multicenter observational study was performed using the eICU Collaborative Research Database (eICU-CRD, version 2.0), which is a public de-identified ICU database comprising 200,859 patient unit encounters for 139,367 unique patients admitted between 2014 and 2015. The eICU database is available from https://physionet.org/content/eicu-crd/. Patients were admitted to 1 of 335 units at 208 hospitals located throughout the United States (Pollard et al., 2018). The eICU-CRD includes data on vital signs, laboratory measurements, medications, Acute Physiology and Chronic Health Evaluation (APACHE) components, care plan information, admission diagnoses, time-stamped diagnoses, and treatments. All researchers of this study received the necessary training and obtained permission to access the database.

### Patient Selection

Adult patients (≥18 years) receiving vancomycin therapy with a single hospital admission and two or more TDM records on the first ICU stay were included in this study. The exclusion criteria were as follows: 1) patients with an ICU length of stay ≤24 h, 2) patients without records of their ICU discharge status, 3) patients with missing or unqualified covariates for multivariable adjustment, and 4) patients with an outlier value of VTC. The upper and lower fences represented values more and less than Q3 and Q1, respectively, by 1.5 times the interquartile range (IQR). The outlier value of VTC was defined as the value above or below the upper (mean VTC >30.70 mg/L) or lower (mean VTC <1.82 mg/L) fences (IQR was calculated using the formula: Q3—Q1).

### Outcomes and Covariates

The outcomes of this study were ICU and hospital mortalities. The variables, mean VTC or serum creatinine (Scr), were calculated by dividing the sum of all collected VTC or Scr by the frequency of monitoring. Mean creatinine clearance (CCl) was determined by the Cockcroft-Gault equation: ([140—age in years] × weight in kg/[72 × mean Scr concentration in mg/dl]) × 0.85 if female (Cockcroft and Gault, 1976). Related treatments (such as dialysis, ventilation, vasodepressor, and vasopressor administration) were included, as these may reflect illness severity and/or affect VTC throughout ICU stay. Variables such as demographic data (e.g., age, sex, and ethnicity), initial body mass index (BMI), APACHE IV score, and comorbidities that could influence mortality (e.g., sepsis, burns, pancreatitis, gastrointestinal bleed, diabetes, heart failure, chronic obstructive pulmonary disease [COPD], hepatic failure, tumour, pneumonia, and renal failure) were also assessed during the first 24 h of ICU admission.

### Statistical Analysis

Based on the recommended VTC in a series of guidelines (Rybak et al., 2009; Matsumoto et al., 2013; Ye et al., 2016), we divided the mean VTC into four categories: <10, 10–15, 15–20, and >20 mg/L. Continuous variables were presented as the medians (IQR) and compared using the Kruskal-Wallis H test. Categorical variables were presented as frequencies (percentages) and compared using χ^2^ or Fisher’s exact tests. A Chord diagram showed the connection between the initial VTC and mean VTC for each patient. Logistic regression models were used to investigate the association of the mean VTC, as a continuous and categorical variable, with ICU and hospital mortalities. To flexibly model and visualise the relationship between mean VTC and mortality, we also used restricted cubic splines with four knots at the fifth, 35th, 65th, and 95th percentiles. Interaction and subgroup analyses were conducted to determine whether the relationship between mean VTC and mortality persisted when the severity of the clinical status (≤64, or >64), CCl (≤80, or >80 ml/min), age (≤60, or >60 years), or BMI (≤30, or >30 kg/m^2^) changed. In addition, the subpopulation with records of vancomycin dose and duration was abstracted for another analysis. All tests were two-sided, and a *p* value < 0.05 was considered statistically significant. Data were extracted using the SAS version 9.4 software (SAS Institute, Cary, NC, United States), and all statistical analyses were performed using SPSS 22.0 (SPSS, Inc., Chicago, IL, United States) and Stata 14.0 (Stata Corp., College Station, TX, United States).

## Results

### Individual Selection and Clinical Characteristics

**FIGURE 1:**
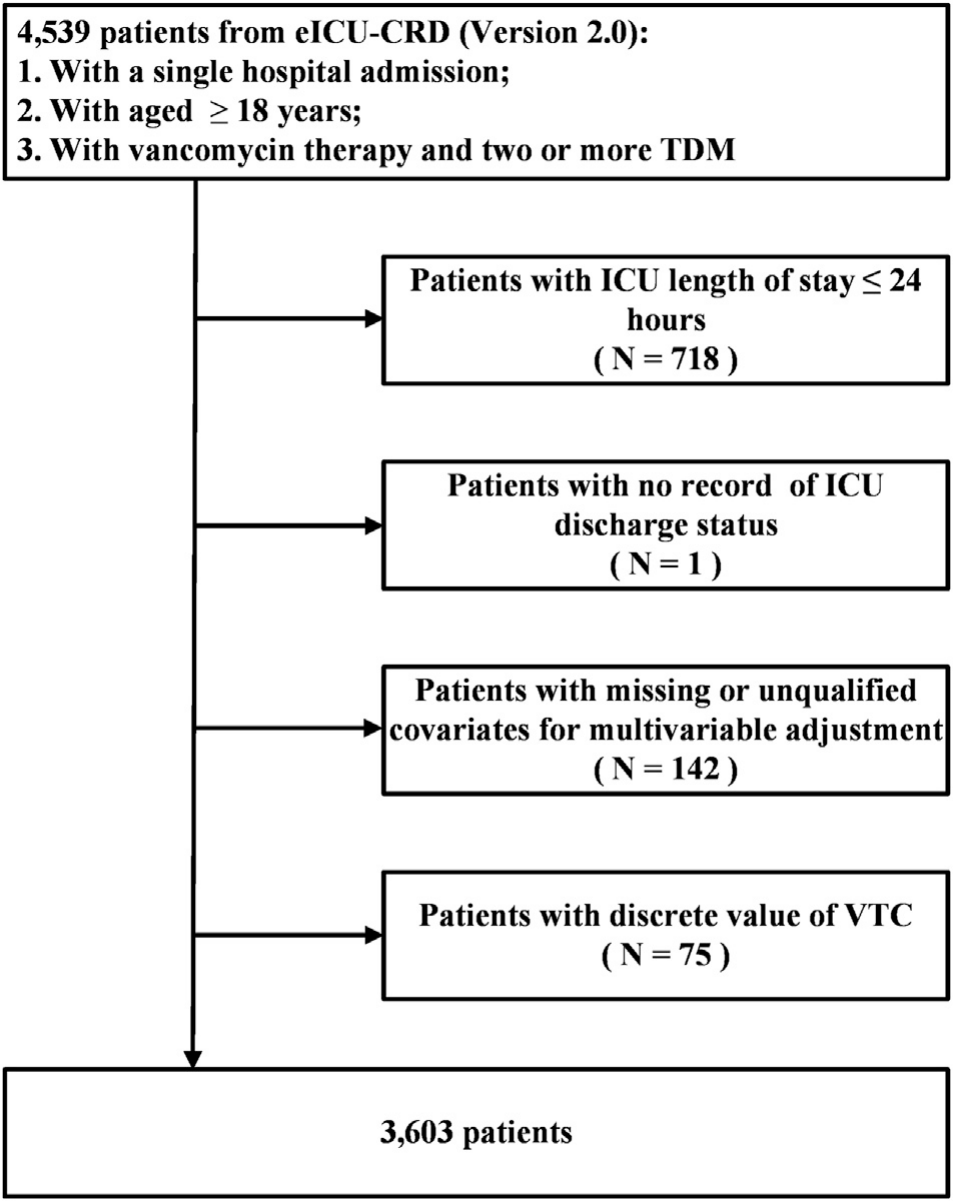
Flow Chart of Participant Selection. Cohort selection and criteria for exclusion, a total of 3,603 patients were included in the analysis. eICU-CRD, eICU Collaborative Research Database; TDM, therapeutic drug monitoring; ICU, intensive care unit; VTC, vancomycin trough concentration.

**FIGURE 2:**
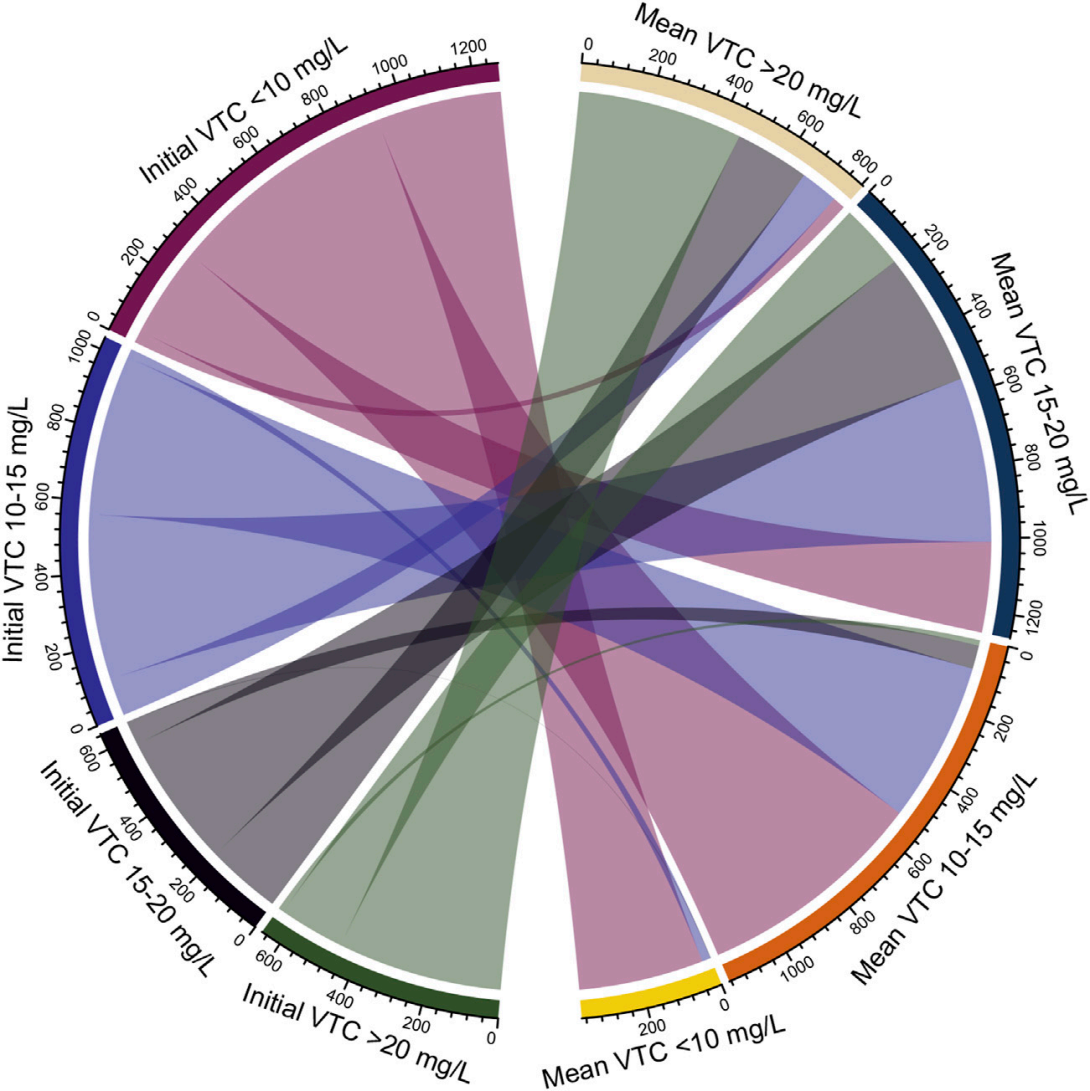
Connection Between the Mean VTC and Initial VTC. A chord diagram presented the difference of the mean VTC with initial VTC for each patient. VTC, vancomycin trough concentration.

A total of 4,539 adult patients with a single hospital stay and at least two VTC records at the first ICU admission were extracted from the eICU-CRD. The followings were excluded: 718 patients with a length of ICU stay ≤24 h, 1 patient without a record of ICU discharge status, 142 patients with missing or unqualified covariates for multivariable adjustment, and 75 patients with a discrete VTC. Finally, 3,603 patients were included in this study (Figure 1). The patients were divided into the following four groups according to the mean VTC during ICU stay: <10 mg/L (*n* = 372, 10.3%), 10–15 mg/L (*n* = 1,165, 32.3%), 15–20 mg/L (*n* = 1,261 35.0%), and >20 mg/L (*n* = 805, 22.3%). Age, BMI, mean VTC, number of VTC, APACHE IV score, Scr, CCl, and use of vasodepressor, vasopressor and dialysis were significantly different among the four groups (*p* < 0.05). Additionally, there was a significant difference in the prevalence of COPD, heart failure, and renal failure (*p* < 0.05). Groups with a higher mean VTC had higher ICU (4.6, 8.2, 11.6, and 15.4%, respectively; *p* < 0.001) and hospital (10.5, 14.1, 18.5, and 22.0%, respectively; *p* < 0.001) mortalities (Table 1). The Chord diagram presented the connection between the mean VTC and initial VTC for each patient (Figure 2). Among all patients, there were 1,622 (45.0%) patients with the mean VTC and the initial VTC in the same groups. 250 (19.7%) patients with initial VTC <10 mg/L had a mean VTC 15–20 mg/L. Among patients with initial VTC 10–15 mg/L, 458 (44.3%) patients reached a mean VTC within 15–20 mg/L. And 182 (27.8%) patients with initial VTC >20 mg/L had a mean VTC 15–20 mg/L.

**TABLE 1:** Baseline characteristics of the study cohort according to mean VTC categories.

Characteristics	Entire population	Mean VTC				
		<10 mg/L	10–15 mg/L	15–20 mg/L	>20 mg/L	*p* Value
	(*N* = 3,603)	(*N* = 372)	(*N* = 1,165)	(*N* = 1,261)	(*N* = 805)	
Age n (%)						<0.001
≤60 years	1,674 (46.5)	209 (56.2)	569 (48.8)	529 (42.0)	367 (45.6%)	
>60 years	1929 (53.5)	163 (43.8)	596 (51.2)	732 (58.0)	438 (54.4%)	
Sex n (%)						0.264
Male	2097 (58.2)	199 (53.5)	690 (59.2)	739 (58.6)	469 (58.3)	
Female	1,506 (41.8)	173 (46.5)	475 (40.8)	522 (41.4)	336 (41.7)	
Ethnicity n (%)						0.303
Caucasian	2,791 (77.5)	277 (74.5)	910 (78.1)	990 (78.5)	614 (76.3)	
Others	812 (22.5)	95 (25.5)	255 (21.9)	271 (21.5)	191 (23.7)	
BMI (kg/m^2^) median (IQR)	27.41 (23.25,33.30)	25.90 (22.25,31.60)	26.81 (23.02,32.55)	27.73 (23.48,33.39)	28.86 (24.09,35.13)	<0.001
Mean VTC (mg/L) median (IQR)	15.90 (12.55,19.57)	8.49 (7.25,9.35)	12.77 (11.50,13.89)	17.17 (16.05,18.46)	22.53 (21.00,24.80)	<0.001
Number of VTC median (IQR)	2 (2,3)	2 (2,3)	2 (2,3)	3 (2,4)	2 (2,3)	<0.001
APACHE IV score median (IQR)	64 (48,83)	58 (41,77)	62 (47,80)	64 (48,84)	69 (53,89)	<0.001
Vasodepressor n (%)	1,743 (48.4)	161 (43.3)	541 (46.4)	615 (48.8)	426 (52.9)	0.006
Vasopressor n (%)	1,318 (36.6)	127 (34.1)	380 (32.6)	494 (39.2)	317 (39.4)	0.002
Ventilation n (%)	2,361 (65.5)	240 (64.5)	732 (62.8)	852 (67.6)	537 (66.7)	0.081
Scr (mg/dl) median (IQR)	0.89 (0.66,1.35)	0.68 (0.52,0.90)	0.77 (0.61,1.10)	0.95 (0.70,1.38)	1.17 (0.83,1.89)	<0.001
CCl (ml/min) median (IQR)	96.57 (59.36,144.69)	129.72 (86.58,182.60)	109.82 (70.33,158.53)	91.87 (57.72,133.30)	75.22 (43.51,119.53)	<0.001
Dialysis n (%)	231 (6.4)	5 (1.3)	47 (4.0)	87 (6.9)	92 (11.4)	<0.001
Diagnoses n (%)						
Tumour	304 (8.4)	28 (7.5)	99 (8.5)	104 (8.2)	73 (9.1)	0.831
Hepatic failure	25 (0.7)	0 (0)	8 (0.7)	11 (0.9)	6 (0.7)	0.124
COPD	302 (8.4)	18 (4.8)	110 (9.4)	125 (9.9)	49 (6.1)	0.001
Heart failure	307 (8.5)	13 (3.5)	85 (7.3)	127 (10.1)	82 (10.2)	<0.001
Diabetes	433 (12.0)	36 (9.7)	149 (12.8)	156 (12.4)	92 (11.4)	0.392
Gastrointestinal bleed	285 (7.9)	38 (10.2)	87 (7.5)	100 (7.9)	60 (7.5)	0.354
Pancreatitis	57 (1.6)	4 (1.1)	20 (1.7)	19 (1.5)	14 (1.7)	0.820
Burns	8 (0.2)	2 (0.5)	3 (0.3)	3 (0.2)	0 (0)	0.183
Pneumonia	950 (26.4)	83 (22.3)	315 (27.0)	345 (27.4)	207 (25.7)	0.237
Sepsis	1,276 (35.4)	114 (30.6)	410 (35.2)	466 (37.0)	286 (35.5)	0.169
Renal failure	510 (14.2)	34 (9.1)	153 (13.1)	192 (15.2)	131 (16.3)	0.005
ICU Mortality n (%)	382 (10.6)	17 (4.6)	95 (8.2)	146 (11.6)	124 (15.4)	<0.001
Hospital mortality n (%)	613 (17.0)	39 (10.5)	164 (14.1)	233 (18.5)	177 (22.0)	<0.001

VTC, vancomycin trough concentration; BMI, body mass index; IQR, interquartile range; APACHE, Acute Physiology and Chronic Health Evaluation; Scr, serum creatinine; CCl, creatinine clearance; COPD, chronic obstructive pulmonary disease; ICU, intensive care unit.

### Association of Mean VTC With Mortality

The univariable logistic regression model revealed that the mean VTC, as a continuous variable, was positively correlated with ICU (odds ratio, 1.073, 95% confidence interval, [1.051–1.095]) and hospital (1.054 [1.036–1.072]) mortalities. This association was still robust (1.038 [1.014–1.063]; 1.025 [1.005–1.046], respectively) after adjusting for age, sex, ethnicity, BMI, APACHE IV score, CCl, the use of ventilation, dialysis, vasodepressor and vasopressor, and diagnoses at ICU admission (Table 2). When mean VTC was considered as a categorical variable, patients with mean VTCs of 10–15, 15–20, and >20 mg/L were associated with higher ICU mortality (1.854 [1.091–3.150]; 2.734 [1.632–4.582]; 3.802 [2.254–6.414]), and those with mean VTCs of 15–20 and >20 mg/L showed a significant increase for hospital mortality (1.935 [1.349–2.776]; 2.407 [1.660–3.489]) compared with those with mean VTC <10 mg/L in the univariable logistic regression analyses. After multivariable adjustment, mean VTC of 10–15 mg/L was not associated with reduced ICU (1.705 [0.975–2.981]) and hospital (1.235 [0.829–1.841]) mortalities. Mean VTC of 15–20 mg/L was not correlated with a lower risk of hospital mortality (1.370 [0.924–2.029]). Moreover, mean VTCs of 15–20 and >20 mg/L were significantly associated with higher ICU mortality (1.924 [1.111–3.332]; 2.428 [1.385–4.258]), and mean VTC of >20 mg/L with increased hospital mortality (1.585 [1.053–2.387]) compared with mean VTC of <10 mg/L (Table 2). Restricted cubic splines visually showed that the risks of ICU (A) and hospital (B) mortalities increased with an increasing mean VTC (Supplementary Figure 1).

**TABLE 2:** Logistic analysis for the association of mean VTC with mortality.

	ICU mortality		Hospital mortality	
VTC variable	OR (95% CI)	*p* value	OR (95% CI)	*p* value
Univariable model				
Continuous variable	1.073 (1.051,1.095)	<0.001	1.054 (1.036,1.072)	<0.001
Categorical variable				
<10 mg/L	1		1	
10–15 mg/L	1.854 (1.091,3.150)	0.022	1.399 (0.966,2.026)	0.076
15–20 mg/L	2.734 (1.632,4.582)	<0.001	1.935 (1.349,2.776)	<0.001
>20 mg/L	3.802 (2.254,6.414)	<0.001	2.407 (1.660,3.489)	<0.001
Multivariable model				
Continuous variable	1.038 (1.014,1.063)	0.002	1.025 (1.005,1.046)	0.012
Categorical variable				
<10 mg/L	1		1	
10–15 mg/L	1.705 (0.975,2.981)	0.061	1.235 (0.829,1.841)	0.299
15–20 mg/L	1.924 (1.111,3.332)	0.019	1.370 (0.924,2.029)	0.117
>20 mg/L	2.428 (1.385,4.258)	0.002	1.585 (1.053,2.387)	0.027

**Multivariable model:** adjusted for age (category), sex, ethnicity, BMI, APACHE IV score, CCl, the use of ventilation, dialysis, vasodepressor and vasopressor, and diagnoses at ICU admission (tumour, hepatic failure, COPD, heart failure, diabetes, gastrointestinal bleed, pancreatitis, burns, pneumonia, sepsis, and renal failure).

VTC, vancomycin trough concentration; ICU, intensive care unit; OR, odds ratio; CI, confidence interval; BMI, body mass index; APACHE, Acute Physiology and Chronic Health Evaluation; CCl, creatinine clearance; COPD, chronic obstructive pulmonary disease.

### Association of Mean VTC With Mortality in Different Subgroups

We further analyzed the association between mean VTC and mortality in different predefined subgroups: APACHE IV score ≤64 (*n* = 1,841) and APACHE IV score >64 (*n* = 1,762); CCl ≤80 ml/min (*n* = 1,393) and CCl >80 ml/min (*n* = 2,210); age ≤60 years (*n* = 1,674) and age>60 years (*n* = 1,929); BMI ≤30 kg/m^2^ (*n* = 2,245) and BMI >30 kg/m^2^ (*n* = 1,358). There was no significant interaction stratified by APACHE IV score (ICU mortality, *p*_interaction_ = 0.545; hospital mortality, *p*_interaction_ = 0.058), CCl (ICU mortality, *p*_interaction_ = 0.807; hospital mortality, *p*_interaction_ = 0.769), age (ICU mortality, *p*_interaction_ = 0.469; hospital mortality, *p*_interaction_ = 0.314) and BMI (ICU mortality, *p*_interaction_ = 0.636; hospital mortality, *p*_interaction_ = 0.627). Notably, mean VTC, as a continuous variable, was significantly correlated with both ICU and hospital mortalities in APACHE IV score >64, CCl ≤80 ml/min, age ≤60 years and BMI ≤30 kg/m^2^ subgroups. When mean VTC was used as a categorical variable, similar results were observed across the different subgroups. Compared with patients with mean VTC of <10 mg/L, those with mean VTCs of 10–15 and 15–20 mg/L were not associated with reduced ICU and hospital mortalities in all subgroups (Table 3; Supplementary Tables 1, 2, 3).

**TABLE 3:** Multivariable analysis for the association of mean VTC with mortality in Apache IV score subgroups.

	ICU mortality		Hospital mortality	
VTC variable	OR (95% CI)	*p* value	OR (95% CI)	*p* value
APACHE IV score ≤64				
Continuous variable	0.995 (0.952,1.040)	0.838	0.980 (0.946,1.015)	0.257
Categorical variable				
<10 mg/L	1		1	
10–15 mg/L	1.486 (0.588,3.758)	0.403	0.857 (0.475,1.546)	0.609
15–20 mg/L	1.443 (0.576,3.618)	0.434	0.803 (0.446,1.449)	0.467
>20 mg/L	1.459 (0.549,3.873)	0.449	0.809 (0.423,1.550)	0.523
APACHE IV score >64				
Continuous variable	1.057 (1.027,1.087)	<0.001	1.049 (1.024,1.074)	<0.001
Categorical variable				
<10 mg/L	1		1	
10–15 mg/L	1.724 (0.864,3.438)	0.122	1.552 (0.916,2.629)	0.102
15–20 mg/L	2.053 (1.043,4.040)	0.037	1.889 (1.125,3.172)	0.016
>20 mg/L	2.895 (1.457,5.751)	0.002	2.333 (1.371,3.970)	0.002

**Multivariable model:** adjusted for age (category), sex, ethnicity, BMI, CCl, the use of ventilation, dialysis, vasodepressor and vasopressor, and diagnoses at ICU admission (tumour, hepatic failure, COPD, heart failure, diabetes, gastrointestinal bleed, pancreatitis, burns, pneumonia, sepsis, and renal failure).

VTC, vancomycin trough concentration; APACHE, Acute Physiology and Chronic Health Evaluation; ICU, intensive care unit; OR, odds ratio; CI, confidence interval; BMI, body mass index; CCl, creatinine clearance; COPD, chronic obstructive pulmonary disease.

We collected 1,553 patients with the records of vancomycin dose and duration to exclude the impact of vancomycin administration on the prognosis of patients. There were significantly statistical differences in total vancomycin dose, average daily dose, and duration of vancomycin exposure across the four groups (*p* < 0.05). The multivariable logistic regression model was built to adjust a series of covariates, including average daily dose and duration of vancomycin exposure, which demonstrated that the relationship between mean VTC and mortality still persisted (Supplementary Table 4).

## Discussion

In this retrospective multicenter cohort study, we recruited a total of 3,603 critically ill patients with two or more VTC monitoring records after vancomycin treatment from 335 different ICUs at 208 hospitals in the eICU-CRD. Mean VTC was calculated by dividing the sum of all collected VTC by the frequency of monitoring for each patient. Our study showed that mean VTCs of 10–15, 15–20, and >20 mg/L were not associated with reduced ICU and hospital mortalities for critically ill patients. Patients with mean VTCs of 15–20 and >20 mg/L were found to be exposed to even higher risks of ICU or hospital mortality compared with those with mean VTC of <10 mg/L. The results indicated that maintaining therapeutic serum VTC between 15 mg/L and 20 mg/L throughout the entire course of vancomycin treatment failed to demonstrate a benefit for ICU and hospital mortalities in ICU patients, and implied that VTC might not be a suitable monitoring indicator to ensure vancomycin efficacy for critically ill patients.

As serum VTC has been suggested as a surrogate marker for the AUC/MIC index to monitor vancomycin efficacy, several studies have been designed to verify the association of VTC with clinical and microbiological outcomes in critically ill patients (Rybak et al., 2009). Surprisingly, most studies found no statistical difference in treatment outcomes according to the VTC level. Two retrospective studies have shown that VTC alone is not a good indicator for vancomycin treatment success among patients with MRSA bacteremia (Jung et al., 2014; Makmor-Bakry et al., 2019). Two other retrospective cohort studies have demonstrated that VTC >15 mg/L fails to improve the outcomes of patients with MRSA infections (Clemens et al., 2011; Hall et al., 2014). Additionally, two prospective, multicenter, observational studies have demonstrated no significant association between VTC level and vancomycin treatment response in a Chinese population diagnosed with gram-positive bacterial infections (Liang et al., 2018; Shen et al., 2018). Moreover, the latest consensus suggests that there are minimal to no data to support the safety and efficacy of a target VTC of 15–20 mg/L in patients with serious MRSA infections (Rybak et al., 2020). However, those aforementioned studies only explored the relationship between single steady-state VTC and outcomes. In a retrospective study of 76 critically ill patients confirmed MRSA infections, *Cheong* reported that the initial VTC was not associated with treatment response, which was consistent with the results of the other studies. Surprisingly, a corrected VTC, calculated as dividing the sum of each measured VTC multiplied by the number of days at that level by the total number of days under treatment, was higher among patients with improved clinical presentation and laboratory results than among those with poor clinical outcomes (Cheong et al., 2012). Because of the lack of large-scale population studies on multiple VTC records after receiving vancomycin therapy, which may represent personalised PK/PD profiles of vancomycin, whether the mean VTC derived from the entire course of therapy is of potential benefit for critically ill patients remains unclear.

In this study, the mean VTC was estimated using all collected VTCs throughout the therapy course, providing an overall level of VTC during the ICU stay. The chord diagram showed 19.7% patients with initial VTC < 10 mg/L, 44.3% patients with initial VTC 10–15 mg/L, and 27.8% patients with initial VTC > 20 mg/L had reached a mean VTC 15–20 mg/L eventually, which indicated that initial sub-therapeutic or excessive VTC had been adjusted to achieve suggested VTC. Therefore, mean VTC could reflect exposure dosage of vancomycin after adjustment for those with lower or higher initial VTC, to some extent. We further found that mean VTCs of 15–20 and >20 mg/L were significantly correlated with higher ICU mortality (1.924-fold and 2.428-fold), mean VTC of >20 mg/L with higher hospital mortality (1.585-fold) than a mean VTC of <10 mg/L. Moreover, mean VTCs of 10–15 and 15–20 mg/L were not associated with reduced ICU/hospital related mortality. Therefore, this present study is one in a growing number of studies demonstrating that maintaining therapeutic VTC might not ensure vancomycin efficacy for critically ill patients. For clinics, our findings suggested that the interpretation of VTC results should be considered cautiously in clinical practice.

The APACHE IV score is useful for assessing the severity of illness and predicting outcomes in ICU patients (Ko et al., 2018; Zimmerman et al., 2006). To diminish the influence of disease severity itself, all individuals were divided into two subgroups for further investigations based on the median first APACHE IV score (≤64 or >64). Vancomycin is eliminated primarily via the renal route, with >80–90% recovered unchanged in urine within 24 h after administration of a single dose (Rybak, 2006). A decrease in the glomerular filtration rate for any cause would increase the VTC, making the association between mortality and VTC difficult to assess (Cantú et al., 1994). To exclude the influence of renal function on outcomes, we classified the patients into two groups according to CCl (≤80 or >80 ml/min) to assess this relationship. Meanwhile, the age and BMI of patient associated with mean VTC can be very significant for mortality (Haeseker et al., 2016; Tsai et al., 2018). Sensitivity analyses were also performed in patients of different ages (≤60 or >60 years) and BMIs (≤30 or >30 kg/m^2^). There is no interaction between mean VTC and those variables for ICU as well as hospital mortalities, which indicated that those variables did not impact the association between mean VTC and mortality. Subgroup analyses also found similar results about the relationships of mean VTC with mortality in all predefined subgroups. Therefore, mean VTC was not associated with improved outcomes regardless of the severity of illness, the degree of renal function, age, or BMI. Based on these results, The AUC/MIC or Bayesian AUC-only estimation may be considered as possible alternative methods for vancomycin administration.

A few underlying mechanisms may explain our results. First, Patel et al. reported that patients with a VTC of 15–20 mg/L could achieve an AUC_0-24_/MIC ratio of ≥400 when the MIC value is ≤ 1 (Patel et al., 2011). However, several studies have demonstrated a poor correlation between VTC and AUC_0–24_ because of high inter-individual variability (Bakke et al., 2017; Haeseker et al., 2016; Neely et al., 2014; Tsai et al., 2018). A 3-years, prospective study indicated that 68% of adults who were administered vancomycin with an AUC_0-24_ ≥ 400 mg h/L had a trough concentration of <15 mg/L (Neely et al., 2018). Additionally, a prospective study of Chinese patients suggested that C_max_, AUC_0–24_, and AUC_0–24 h_/MIC are not significantly associated with clinical and microbiological outcomes based on multivariable logistic regression analysis (Shen et al., 2018). Second, vancomycin has been associated with nephrotoxicity, which is the most serious common side effect of vancomycin linked to a higher risk of mortality (Jeffres, 2017). A multicenter prospective clinical trial including 288 adult patients indicated that a VTC of >15 mg/L is associated with a 3-fold increased risk of nephrotoxicity (Bosso et al., 2011). Another retrospective study in the 315 vancomycin-treated patients observed that vancomycin nephrotoxicity is independently correlated with higher VTC of >20 mg/L (Park et al., 2018). The result is similar to those of our study, in which mean VTC >15 mg/L was associated with increased ICU or hospital mortality. Therefore, the optimal TDM of vancomycin in ICU patients warrants further investigation.

Our study has some limitations. First, given the retrospective nature of the study, the risk of unmeasured confounding effects and the introduction of bias were unavoidable. However, this multicenter study of VTC included the largest sample size to date, allowing the findings to be generalised. Second, there is no available information on microbiological outcomes and acute kidney injury after vancomycin treatment in eICU-CRD. Nevertheless, mortality may represent clinical outcomes to confirm the prognostic value of VTC, as critically ill patients with gram-positive bacterial infection have high mortality, particularly owing to MRSA infections (Calfee et al., 2014). Third, hospital protocols for the time of VTC monitoring, target VTC, and adjustments of vancomycin were not available due to the retrospective design. However, the sensitivity analysis found the association was persisted in less than half of the patients with records on vancomycin dose and duration. Fourth, we can provide only the association between mean VTC and mortality rather than causality. In the future, a well-designed randomised controlled trial with more treatment information should be considered to evaluate causality between VTC and mortality.

## Conclusion

In conclusion, mean VTC was not associated with reduced ICU/hospital related mortality, independent of the degree of disease severity, renal function, age, and BMI. Our results indicated that VTC monitoring might not ensure vancomycin efficacy for ICU patients. Therefore, the interpretation of VTC results should be considered cautiously for clinics in clinical practice. The AUC/MIC or Bayesian AUC-only estimation may be considered as possible alternative methods for vancomycin administration, and warrant further investigations in future studies.

## Data Availability

Publicly available datasets were analyzed in this study. This data can be found here: https://eicu-crd.mit.edu.
