# Supplementary material for: Relationship Between Mean Vancomycin Trough Concentration and Mortality in Critically Ill Patients: A Multicenter Retrospective Study
Source: Front Pharmacol. 2021 Jul 19;12:690157. doi: 10.3389/fphar.2021.690157 (PMC8326564; doi:10.3389/fphar.2021.690157)
Supplement: Supplementary file 1 [file Table1.DOCX]

**Additional File 1: Fig. 1** Association of mean VTC with ICU (A) and hospital (B) mortalities.

Restricted cubic splines reveal that the risks of ICU (A) and hospital (B) mortalities increased with an increase in mean VTC. All models were adjusted for age (category), sex, ethnicity, BMI, APACHE IV score, CCl, the use of ventilation, dialysis, vasodepressor and vasopressor, and diagnoses at ICU admission (tumour, hepatic failure, COPD, heart failure, diabetes, gastrointestinal bleed, pancreatitis, burns, pneumonia, sepsis, and renal failure)

VTC, vancomycin trough concentration; ICU, intensive care unit; OR, odds ratio; CI, confidence interval; BMI, body mass index; APACHE, Acute Physiology and Chronic Health Evaluation; CCl, creatinine clearance; COPD, chronic obstructive pulmonary disease.

**Additional File 2: Table 1. Multivariable Analysis for the Association of Mean VTC with Mortality in CCl Subgroups**

|  | **ICU Mortality** | | **Hospital Mortality** | |
| --- | --- | --- | --- | --- |
| **VTC Variable** | **OR (95% CI)** | ***P* Value** | **OR (95% CI)** | ***P* Value** |
| **CCl ≤80ml/min** |  |  |  |  |
| Continuous variable | 1.048 (1.016,1.081) | 0.003 | 1.031 (1.004,1.059) | 0.024 |
| Categorical variable |  |  |  |  |
| <10 mg/L | 1 |  | 1 |  |
| 10–15 mg/L | 1.944 (0.807,4.680) | 0.138 | 1.005 (0.538,1.877) | 0.986 |
| 15–20 mg/L | 2.263 (0.963,5.318) | 0.061 | 1.269 (0.694,2.321) | 0.439 |
| >20 mg/L | 3.062 (1.296,7.234) | 0.011 | 1.426 (0.773,2.631) | 0.256 |
| **CCl >80ml/min** |  |  |  |  |
| Continuous variable | 1.028 (0.991,1.067) | 0.143 | 1.019 (0.989,1.050) | 0.209 |
| Categorical variable |  |  |  |  |
| <10 mg/L | 1 |  | 1 |  |
| 10–15 mg/L | 1.592 (0.768,3.301) | 0.211 | 1.417 (0.838,2.396) | 0.193 |
| 15–20 mg/L | 1.837 (0.889,3.794) | 0.101 | 1.417 (0.835,2.404) | 0.197 |
| >20 mg/L | 1.951 (0.894,4.258) | 0.093 | 1.646 (0.925,2.930) | 0.090 |

**Multivariable model:** adjusted for age (category), sex, ethnicity, BMI, APACHE IV score, the use of ventilation, dialysis, vasodepressor and vasopressor, and diagnoses at ICU admission (tumour, hepatic failure, COPD, heart failure, diabetes, gastrointestinal bleed, pancreatitis, burns, pneumonia, sepsis, and renal failure)

VTC, vancomycin trough concentration; CCl, creatinine clearance; ICU, intensive care unit; OR, odds ratio; CI, confidence interval; BMI, body mass index; APACHE, Acute Physiology and Chronic Health Evaluation; COPD, chronic obstructive pulmonary disease.

**Additional File 2: Table 2. Multivariable Analysis for the Association of Mean VTC with Mortality in Age Subgroups**

|  | **ICU Mortality** | | **Hospital Mortality** | |
| --- | --- | --- | --- | --- |
| **VTC Variable** | **OR (95% CI)** | ***P* Value** | **OR (95% CI)** | ***P* Value** |
| **Age ≤ 60 y** |  |  |  |  |
| Continuous variable | 1.058 ( 1.018, 1.099) | 0.004 | 1.050 (1.016, 1.084) | 0.003 |
| Categorical variable |  |  |  |  |
| <10 mg/L | 1 |  | 1 |  |
| 10–15 mg/L | 1.320 (0.544, 3.202) | 0.539 | 1.226 (0.620, 2.423) | 0.558 |
| 15–20 mg/L | 1.967 (0.825, 4.689) | 0.127 | 1.622 (0.824, 3.192) | 0.161 |
| >20 mg/L | 2.579 (1.069, 6.224) | 0.035 | 2.178 (1.092, 4.347) | 0.027 |
| **Age > 60 y** |  |  |  |  |
| Continuous variable | 1.025 (0.994, 1.057) | 0.117 | 1.012 (0.987, 1.037) | 0.363 |
| Categorical variable |  |  |  |  |
| <10 mg/L | 1 |  | 1 |  |
| 10–15 mg/L | 1.987 (0.958, 4.121) | 0.065 | 1.229 (0.746, 2.025) | 0.417 |
| 15–20 mg/L | 1.942 (0.948, 3.978) | 0.070 | 1.265 (0.775, 2.066) | 0.348 |
| >20 mg/L | 2.385 (1.141, 4.984) | 0.021 | 1.340 (0.800, 2.243) | 0.266 |

**Multivariable model:** adjusted for sex, ethnicity, BMI, APACHE IV score, CCl, the use of ventilation, dialysis, vasodepressor and vasopressor, and diagnoses at ICU admission (tumour, hepatic failure, COPD, heart failure, diabetes, gastrointestinal bleed, pancreatitis, burns, pneumonia, sepsis, and renal failure)

VTC, vancomycin trough concentration; ICU, intensive care unit; OR, odds ratio; CI, confidence interval; BMI, body mass index; APACHE, Acute Physiology and Chronic Health Evaluation; CCl, creatinine clearance; COPD, chronic obstructive pulmonary disease.

**Additional File 2: Table 3. Multivariable Analysis for Association of Mean VTC with Mortality in BMI Subgroups**

|  | **ICU Mortality** | | **Hospital Mortality** | |
| --- | --- | --- | --- | --- |
| **VTC Variable** | **OR (95% CI)** | ***P* Value** | **OR (95% CI)** | ***P* Value** |
| **BMI ≤ 30 kg/m^2^** |  |  |  |  |
| Continuous variable | 1.046 (1.015,1.079) | 0.003 | 1.032 (1.007,1.058) | 0.012 |
| Categorical variable |  |  |  |  |
| <10 mg/L | 1 |  | 1 |  |
| 10–15 mg/L | 1.982 (0.997,3.940) | 0.051 | 1.197 (0.754,1.901) | 0.446 |
| 15–20 mg/L | 2.337 (1.192,4.582) | 0.013 | 1.468 (0.933,2.309) | 0.097 |
| >20 mg/L | 2.887 (1.438,5.794) | 0.003 | 1.704 (1.054,2.755) | 0.030 |
| **BMI > 30 kg/m^2^** |  |  |  |  |
| Continuous variable | 1.024 (0.986, 1.064) | 0.219 | 1.013 (0.980,1.046) | 0.442 |
| Categorical variable |  |  |  |  |
| <10 mg/L | 1 |  | 1 |  |
| 10–15 mg/L | 1.187 (0.444,3.174) | 0.732 | 1.320 (0.591,2.951) | 0.499 |
| 15–20 mg/L | 1.247 (0.473,3.283) | 0.655 | 1.148 (0.516,2.555) | 0.735 |
| >20 mg/L | 1.597 (0.607,4.202) | 0.343 | 1.375 (0.614,3.077) | 0.439 |

**Multivariable model:** adjusted for age (category), sex, ethnicity, APACHE IV score, CCl, the use of ventilation, dialysis, vasodepressor and vasopressor, and diagnoses at ICU admission (tumour, hepatic failure, COPD, heart failure, diabetes, gastrointestinal bleed, pancreatitis, burns, pneumonia, sepsis, and renal failure)

VTC, vancomycin trough concentration; BMI, body mass index; ICU, intensive care unit; OR, odds ratio; CI, confidence interval; APACHE, Acute Physiology and Chronic Health Evaluation; CCl, creatinine clearance; COPD, chronic obstructive pulmonary disease.

**Additional File 2: Table 4. Sensitivity Analysis for the Association of Mean VTC with Mortality in the Subpopulation with Records of Vancomycin Dose and Duration**

|  | **ICU Mortality** | | **Hospital Mortality** | |
| --- | --- | --- | --- | --- |
| **VTC Variable** | **OR (95% CI)** | ***P* Value** | **OR (95% CI)** | ***P* Value** |
| **Univariable Model** |  |  |  |  |
| Continuous variable | 1.051 (1.019,1.085) | 0.002 | 1.050 (1.023,1.079) | <0.001 |
| Categorical variable |  |  |  |  |
| <10 mg/L | 1 |  | 1 |  |
| 10–15 mg/L | 2.156 (0.997,4.664) | 0.051 | 1.772 (0.986,3.185) | 0.056 |
| 15–20 mg/L | 2.497 (1.166,5.347) | 0.019 | 2.181 (1.226,3.880) | 0.008 |
| >20 mg/L | 3.755 (1.745,8.078) | 0.001 | 2.865 (1.592,5.154) | <0.001 |
| **Multivariable Model** |  |  |  |  |
| Continuous variable | 1.005 (0.968,1.043) | 0.812 | 1.010 (0.979,1.042) | 0.535 |
| Categorical variable |  |  |  |  |
| <10 mg/L | 1 |  | 1 |  |
| 10–15 mg/L | 2.035 (0.856,4.836) | 0.108 | 1.424 (0.746,2.716) | 0.284 |
| 15–20 mg/L | 1.746 (0.745,4.175) | 0.197 | 1.321 (0.694,2.514) | 0.397 |
| >20 mg/L | 2.188 (0.909,5.266) | 0.080 | 1.570 (0.807,3.957) | 0.184 |

**Multivariable model:** adjusted for age (category), sex, ethnicity, BMI, APACHE IV score, CCl, the use of ventilation, dialysis, vasodepressor and vasopressor, average daily dose, duration of vancomycin, and diagnoses at ICU admission (tumour, hepatic failure, COPD, heart failure, diabetes, gastrointestinal bleed, pancreatitis, burns, pneumonia, sepsis, and renal failure)

VTC, vancomycin trough concentration; ICU, intensive care unit; OR, odds ratio; CI, confidence interval; BMI, body mass index; APACHE, Acute Physiology and Chronic Health Evaluation; CCl, creatinine clearance; COPD, chronic obstructive pulmonary disease.
